# Extensive Subcutaneous Emphysema Associated With Pneumothorax, Pneumomediastinum, and Pneumoperitoneum: A Case Report

**DOI:** 10.7759/cureus.31816

**Published:** 2022-11-23

**Authors:** Emmanuel Robinson, Klara Schwarzova, Jacqueline Pursel, Gavin Henry

**Affiliations:** 1 General Surgery, Ascension St. Agnes Hospital, Baltimore, USA; 2 Thoracic Surgery, University of Maryland Medical Center, Baltimore, USA

**Keywords:** subcutaneous emphysema management, pneumoperitoneum, pneumomediastinum, pneumothorax, subcutaneous emphysema

## Abstract

Subcutaneous emphysema (SE ) is a phenomenon in which air occupies structures under the skin and soft tissues. Common sites for SE include the neck and chest wall, which can extend to other body regions. In this case report, we describe the development of extensive SE, pneumothorax, pneumomediastinum, and pneumoperitoneum in an elderly female following blunt trauma to her right flank.

The etiology of SE is broad and includes blunt and penetrating traumas; surgical, infectious, spontaneous causes; or any condition that yields a gradient between intra-alveolar and perivascular interstitial pressures. The incidence of SE has been reported to be 1.4%, while that of spontaneous pneumothorax has been reported to be 0.8% in patients receiving a percutaneous tracheostomy. Conversely, the occurrence of SE, pneumothorax, pneumomediastinum, and pneumoperitoneum in the same patient is rare. The most common signs and symptoms of SE are neck swelling and chest pain. Involvement of the deeper tissues of the thoracic outlet, chest, and abdominal wall often manifests in severe life-threatening conditions. SE can be diagnosed by detecting edema and crepitus of the scalp, neck, thorax, abdomen, and other body regions. Radiograph imaging can confirm the presence of soft-tissue air entry. Extensive SE in the setting of pneumothorax is an unusual entity for which there is, as of now, no consensus in management. Methods of treatment include supportive care, placement of blow holes for evacuation of soft-tissue emphysema, and bilateral infraclavicular incisions. SE is a rare complication that can arise from several etiologies. At the same time, various methods for managing this phenomenon have been mentioned with varying successful outcomes.

## Introduction

Subcutaneous emphysema (SE) is a phenomenon in which air infiltrates structures under the skin and soft tissues. Common sites for SE include the neck and chest wall, which can extend to other body regions. Clinical severity often corresponds to the degree of air leak from the lungs, chest wall, and other hollow viscera. In this case report, we describe the development of extensive SE, pneumothorax, pneumomediastinum, and pneumoperitoneum in an elderly female following blunt trauma to her upper back and right flank. This case report aims to bolster the awareness of SE. While several methods for managing this phenomenon have been mentioned with varying successful outcomes, an extensive review of the literature reveals that there is no consensus among clinicians.

## Case presentation

A 100-year-old female with a medical history significant for stage II chronic kidney disease and hypertension presented to the Emergency Department due to a 36-hour history of diffuse swelling. The patient reported having an acute onset of swelling initially in her chest, which propagated to her head, abdomen, and lower extremities. The patient reported hitting her right flank and upper back against a countertop two days before the presentation. The patient denied any chest pain, increased work of breathing, exertional dyspnea, or any systemic signs of infection such as fever, night sweats, or chills. In addition, she denied any instrumentalization of her chest, recent procedures, or any history of recent retching or vomiting since the onset of her symptoms.

**Figure 1:**
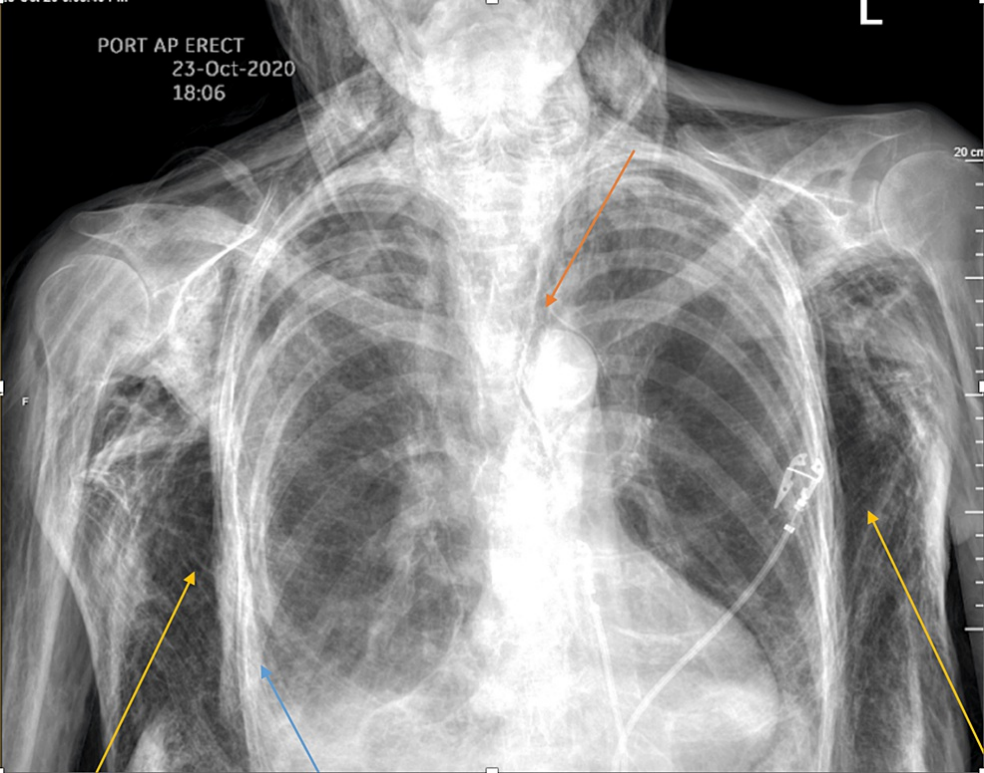
Posteroanterior upright chest X-ray. Findings notable for extensive soft-tissue subcutaneous emphysema (yellow arrows), pneumomediastinum (orange arrow), and small right hydropneumothorax (blue arrow).

**Figure 2:**
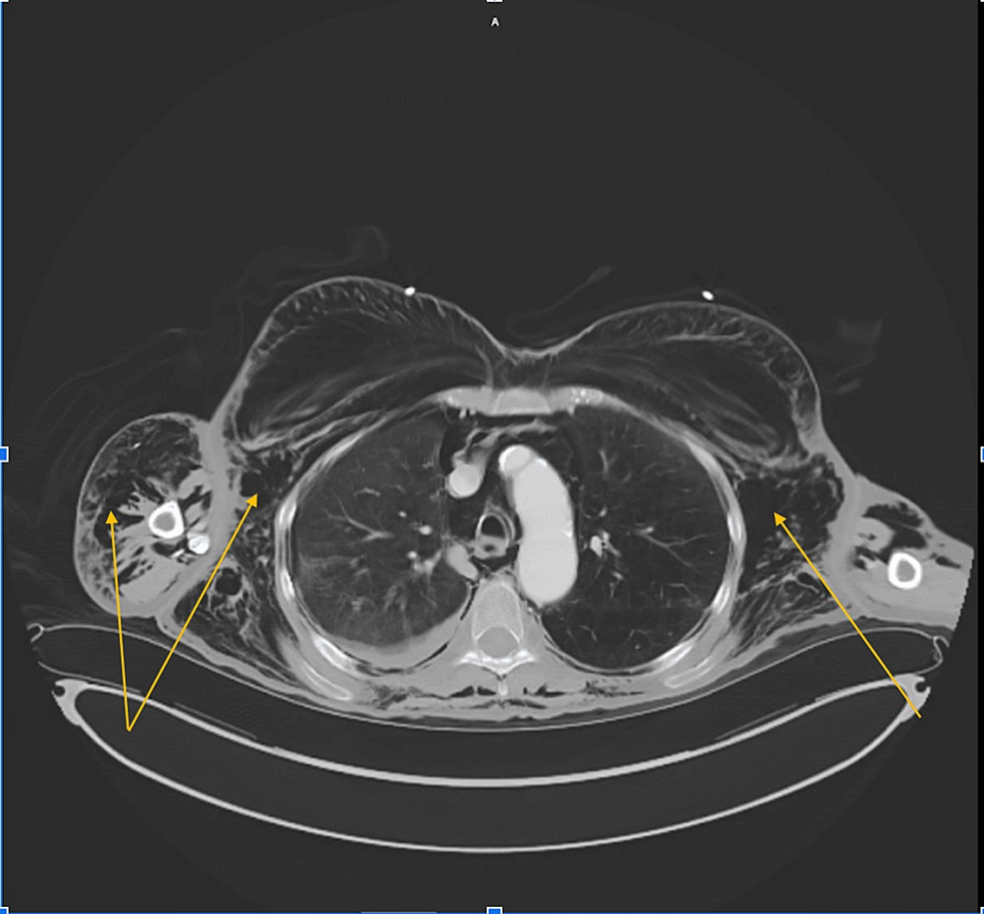
Computed tomography (CT) of the chest/abdomen/pelvis with intravenous contrast - axial view CT abdomen/pelvis in the lung window. The image displays extensive soft-tissue subcutaneous emphysema (yellow arrows).

**Figure 3:**
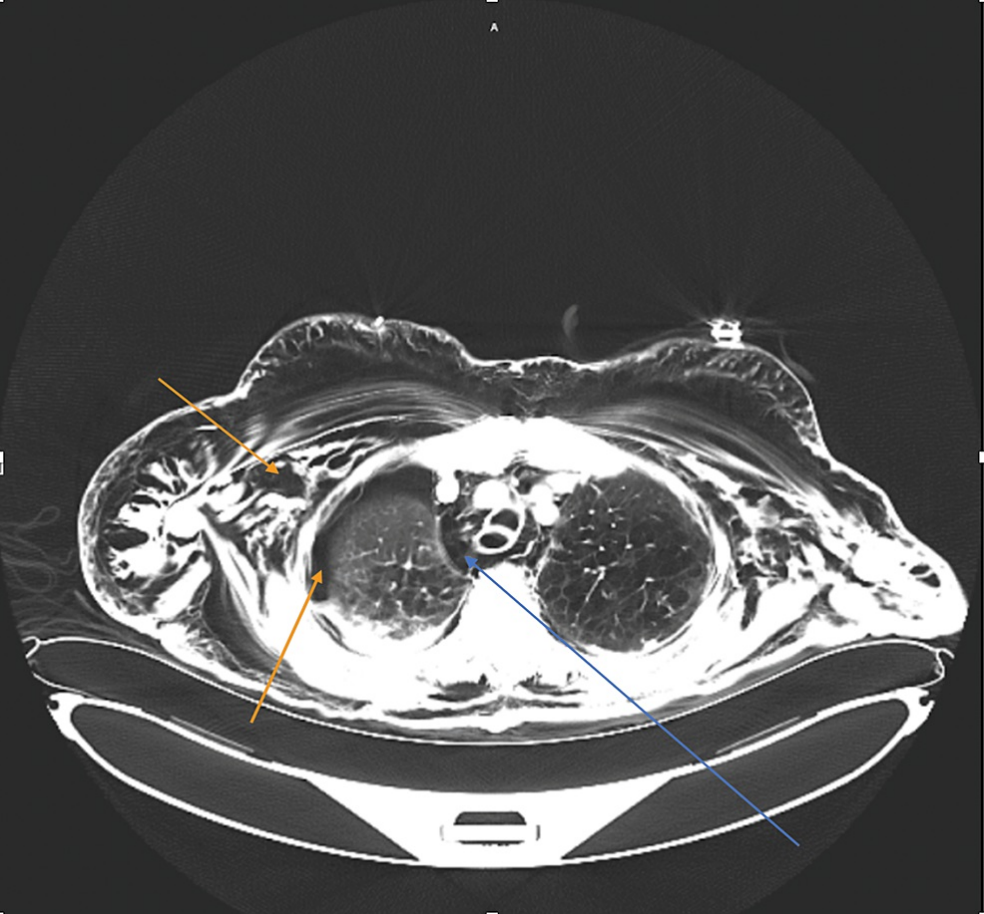
Computed tomography (CT) abdomen/pelvis (A/P) with intravenous contrast - axial view CT A/P in the regular window. Evident is the presence of extensive subcutaneous emphysema (yellow arrow), pneumomediastinum (blue arrow), and a right-sided pneumothorax (orange arrow).

On presentation, the patient was in no acute distress. Vital signs were notable for tachycardia in the low 100s and elevated systolic blood pressure in the 180s. However, the patient maintained oxygen saturation above 95% on room air. Physical exam showed diffuse soft-tissue swelling and emphysematous changes throughout the body, especially remarkable on the head where diffuse SE was surrounding bilateral orbits as well as in the forehead and the maxillary region. The patient was able to phonate, and the extraocular motion was intact. Chest examination showed decreased breath sound on the right lung fields and extensive SE throughout the chest. No tenderness was present on chest palpation. Chest X-ray (CXR) (Figure 1) and computed tomography (CT) A/P (Figures 2, 3) findings were consistent with extensive SE throughout the soft tissues of the visualized neck, chest, abdomen, pelvis, and upper and lower extremities, as well as right-sided pneumothorax. In addition, there was free air in the abdomen and pelvis.

The patient underwent urgent right thoracostomy tube placement and was admitted for further treatment. The chest tube was kept to wall suction for the first few days due to persistent air leaks. Subsequently, the chest tube was put under a water seal, clamped, and removed on hospital day (HD) 6. The patient was discharged to subacute rehab on HD 7. The patient’s hospital course was uncomplicated, and she recovered well.

## Discussion

The etiology of SE is broad and includes blunt and penetrating traumas; surgical, infectious, spontaneous causes; or any condition that yields a gradient between intra-alveolar and perivascular interstitial pressures [1-3]. One case study found the incidence of SE to be 1.4%, while that of spontaneous pneumothorax was reported to be 0.8% in patients receiving percutaneous tracheostomy [4]. Conversely, the occurrence of SE, pneumothorax, pneumomediastinum, and pneumoperitoneum in the same patient is rare [4]. Although several such case reports exist in the literature, the incidence of this clinical condition is lacking [4-7].

The most common signs and symptoms of SE are neck swelling and chest pain [5]. Additional symptoms may include sore throat, dysphagia, breathlessness, wheezing, and abdominal distension [5,8,9]. Involvement of the deeper tissues of the thoracic outlet, chest, and abdominal wall often manifests in severe life-threatening conditions. It can be complicated by restriction of full lung re-expansion and can lead to high airway pressure, severe respiratory acidosis, ventilator failure, pacemaker malfunction, airway compromise, and tension phenomena [5,4].

SE can be diagnosed clinically by the detection of edema and crepitus of the scalp, eyelid, neck, thorax, abdomen, and other body regions. Radiograph imaging, along with chest roentgenogram [8] and CT, can confirm the presence of soft-tissue air entry [5].

Extensive SE in low-impact trauma without associated rib fractures is an unusual entity for which there is, as of now, no consensus in management. One proposed option is placing blow holes [1], circular incisions with partial skin removal to evacuate soft-tissue emphysema [10,11]. This treatment, however, is mainly symptomatic as the source communication between the pleural cavity and surrounding soft tissue is nearly never addressed.

Another management proposal in the literature suggests using bilateral infraclavicular incisions, which are modified blow holes of linear shape to provide better lung expansion and effective air evacuation [5]. Furthermore, this method is thought to provide a rapid and cosmetically appealing outcome compared with more invasive alternatives such as cervical mediastinotomy [5]. Our management largely focused on the right pneumothorax evacuation [12]. The patient’s SE continued to improve throughout her hospital stay, and she did not require blow holes or infraclavicular incisions.

## Conclusions

SE is a rare complication that can arise from several etiologies. Various methods for managing this phenomenon have been mentioned with varying successful outcomes. In this case study, thoracostomy tube placement was viable, safe, and effective. This case study highlights the importance of recognizing frailty in older adults who can develop a traumatic pneumothorax and associated SE even in low-impact injury and absence of rib fractures. To date, there is no consensus on the treatment of choice for SE, and thus more studies are needed.
